# Celecoxib in Treatment of Postpartum Depression: A Case Report

**DOI:** 10.34172/aim.2023.42

**Published:** 2023-05-01

**Authors:** Sophia Esalatmanesh, Ladan Kashani, Shahin Akhondzadeh

**Affiliations:** ^1^Arash Women’s Hospital, Tehran University of Medical Sciences, Tehran, Iran; ^2^Psychiatric Research Center, Roozbeh Hospital, Tehran University of Medical Sciences, Tehran, Iran

**Keywords:** Case report, Celecoxib, Postpartum depression

## Abstract

Postpartum depression (PPD) impairs mother-infant interaction and has negative effects on the child’s emotional, behavioral, and cognitive skills. There is considerable evidence to suggest that inflammation plays a role in the pathogenesis of depression. Controlled trials indicate that celecoxib has antidepressant effects in patients with major depressive disorder. A 34-year-old woman with mild to moderate PPD received a celecoxib capsule twice a day. This treatment has not been reported in previous studies and is novel in clinical practice. The patient was assessed using the Hamilton Depression Rating Scale (HDRS). Moreover, levels of brain-derived neurotrophic factor (BDNF) and inflammatory cytokines were measured at baseline and at the end of celecoxib therapy. This case suggests that celecoxib can improve depressive symptoms in patients with mild to moderate PPD. No adverse effects occurred during follow-up.

## Introduction

 Postpartum depression (PPD) is defined as a subtype of major depressive disorder if the onset of symptoms occurs within 4 weeks after delivery.^1^ Prevalence of PPD is 17% in healthy mothers.^2^ Children of women with PPD are at increased risk of emotional, behavioral, and cognitive problems.^3^ Several mechanisms have been considered for PPD. Steroid hormones play an important role in PPD. The levels of estradiol rise during pregnancy and decrease dramatically after delivery, leading to the estradiol-withdrawal state hypothesis associated with PPD.^4^ This significant fluctuation after delivery is related to decreased mood, which is associated with increased levels of the serotonin transporter in the neocortex, resulting in decreased serotonin levels and depression symptoms.^5^ In addition, depression is associated with changes in the hippocampus, amygdala, and prefrontal cortex.^6^ Psychotherapy and antidepressant medications are used as main treatment for PPD.^7^ Depression is a neuroplastic disorder. Stressors and cytokines can destroy nerve plasticity by altering neurotransmitters that contribute to depression.^8^ Here, we present a successful and uncomplicated treatment with celecoxib in a patient with mild to moderate PPD.

## Case Report

 A 33-year-old woman, on the 17th day of her postpartum period, was referred to the psychiatric clinic of Roozbeh hospital (affiliated with Tehran University of Medical Sciences, Tehran, Iran) due to depressive symptoms. In her psychiatric history, she had two children. Her first pregnancy was at the age of 27 and she did not mention any previous episodes of major depressive disorder. Her recent pregnancy was normal without any history of depression or medical problems related to her pregnancy and her family support was good. She complained of sadness, helplessness, and ideas of guilt over her past errors. She was worried about taking care of her children. Furthermore, she had initial insomnia and loss of appetite and described feeling exhausted and decreased motivation for carrying out her duties at home. Psychomotor agitation, disorganized behavior, ideo-verbal dissociation, hallucinatory delirium, and aggression were not found. She did not have any suicidal or infanticidal thoughts, history of bipolar disorder, or substance or alcohol dependence. She did not breastfeed. Moreover, the patient did not have hypothyroidism, cardiovascular disease, gastrointestinal bleeding, or any acute medical illness in her medical history. Physical examination and electrocardiography revealed no abnormalities. Laboratory findings were within normal limits. Our patient was assessed using the Hamilton Depression Rating Scale (HDRS) 17-item version which contains 17 questions (on a three-point or five-point scale). This scale has been applied in many clinical trials to explore severity of depression.^9,10^ According to the HDRS, she had a score of 16.

 After consultation, according to the positive effects of celecoxib on improvement of depressive symptoms in many clinical trials,^11,12^ treatment was started with celecoxib (Celexib, Daroopakhsh, 200 mg capsules) twice a day as outpatient therapy. Psychiatric visits were performed every two weeks and comprehensive history was taken at every visit during therapy. The adverse effects of celecoxib were described for the patient and she was asked to immediately inform her psychiatrist about any unexpected complications and suicidal or infanticidal thoughts during therapy. Also, maternal non-fasting blood samples were taken from patients at baseline and at the end of celecoxib therapy. Samples were centrifuged at 850 g for 20 minutes at 4 °C and serum was stored at −80 °C until analysis. Serum was analyzed using a commercially available enzyme immunoassay kit (DuoSet ELISA Development, R&D Systems Inc., USA) to evaluate levels of brain-derived neurotrophic factor (BDNF), tumor necrosis factor-α (TNF-α), interferon-γ (IFN-γ), interleukin-1β (IL-1β), IL-6, IL-8, erythrocyte sedimentation rate (ESR) and C-reactive protein (CRP).

 The patient’s mood, appetite, and functional state improved markedly four weeks after beginning treatment. During the 6 weeks following initiation of celecoxib therapy, the patient’s depressive symptoms improved dramatically from 16 to 3 according to the HDRS. Table 1 shows levels of laboratory markers at baseline and at 6 weeks after beginning treatment. Levels of ESR, CRP, TNF-α, IL-1β, IL-6 and IL-8 decreased at week 6. Also, levels of BDNF increased at week 6. No serious adverse effect occurred during our follow-up.

**Table 1:** Laboratory Markers Results

**Variable**	**Baseline**	**Week 6, After Celecoxib Therapy**
ESR (mm/h)	55.00	35.00
CRP (mg/L)	1.00	0.50
TNF-α (pg/mL)	2.20	1.70
IFN-γ (pg/mL)	0.97	0.97
Il-1β (pg/mL)	2.00	1.80
IL-6 (pg/mL)	2.30	1.20
IL-8 (pg/mL)	3.50	3.00
BDNF (ng/mL)	24.00	26.00

BDNF, brain-derived neurotrophic factor; IFN-γ, interferon-γ; TNF-α, tumor necrosis factor-α; ESR, erythrocyte sedimentation rate; CRP, C-reactive protein; IL-1β, interleukin-1β.

## Discussion

 A growing body of evidence indicate that inflammatory processes have a key role in the pathophysiology of depression.^13^ Depression is associated with an increase in inflammation and hyperactivity of the hypothalamic-pituitary-adrenocortical (HPA) axis^14^ and inflammatory disease is related to depression.^8^ Moreover, administration of immunomodulating agents like interferon-α (IFN-α) in patients with malignant melanoma^15^ can induce depressive symptoms. Previous studies have shown that increased levels of proinflammatory cytokines are seen in depressed patients.^16^ Also, there is a linear relationship between severity of depression and concentration of inflammatory cytokines, including IL-1β, IL-8, TNF-α,^17^ and IL-6.^18^ BDNF levels in women with PPD are low.^19^ A marked decrease in serum BDNF concentration occurs both before and after delivery.^20^ Antidepressant treatments increase neural plasticity by activating BDNF.^21^ Yoshimura et al indicated a negative correlation between serum BDNF levels and baseline HDRS scores. In addition, BDNF levels were significantly increased 8 weeks after treatment with paroxetine or milnacipran in responders.^22^

 Antidepressants can reduce serum levels of inflammatory cytokines and regulate cytokine actions by acting on intracellular adenosyl monophosphate (cAMP), serotonin metabolism, HPA axis, or neurogenesis.^23^ Another mechanism that has been considered for inflammation-induced depression is alteration of glutamatergic neurotransmission.TNF-α and interferon-γ stimulate activation of indoleamine 2, 3 dioxygenase (IDO). This enzyme is produced by macrophages and microglia and can produce neuroactive tryptophan metabolites which induce depression-like behavior.^24^ Cyclooxygenase-2 (COX-2) induces production of prostaglandins in inflammation.^25^ The effect of celecoxib as a selective COX-2 inhibitor in treatment of depression has been widely studied in numerous studies. A clinical trial was carried out to evaluate the effect of celecoxib as monotherapy in treatment of mild to moderate depression in patients with colorectal cancer for 6 weeks. The results of this study showed that patients who received celecoxib had significant improvement in HDRS score than the placebo arm at the end of the study (*P* = 0.003).^26^ In a six-week clinical trial, levels of IL-6 were measured in patients who received celecoxib or a placebo in addition to sertraline. The results indicated a significantly greater reduction in serum levels of IL-6 (*P* < 0.001) as well as HDRS score (*P* = 0.005) in the celecoxib group. On the other hand, a significant correlation was found between the reduction of HDRS scores and reduction in IL-6 levels at the endpoint of this trial.^15^ Another study demonstrated that patients with depressive or mixed episodes of bipolar disorder who received celecoxib had lower HDRS scores compared to the placebo group.^10^ Jafari et al emphasized that administration of 200 mg bid of celecoxib for 8 weeks had a significant effect in treatment of depressed patients with acute brucellosis and no serious adverse effects were observed in the celecoxib group.^27^ Results of a systematic review demonstrated that celecoxib was an effective add-on treatment for unipolar depression.^11^ Another study revealed that levels of TNF-α and CRP were significantly lower compared to the control group with antidepressant treatment (*P* < 0.001).^28^

 This case report suggests that celecoxib may be a therapeutic option for treatment of mild to moderate PPD. No adverse effect related to celecoxib occurred during our follow-up. However, further clinical trial studies are needed to evaluate the efficacy and safety of celecoxib in treatment of PPD.
